# Supplementary material for: Ticagrelor alleviates pyroptosis of myocardial ischemia reperfusion-induced acute lung injury in rats: a preliminary study
Source: PeerJ. 2024 Jan 4;12:e16613. doi: 10.7717/peerj.16613 (PMC10771767; doi:10.7717/peerj.16613)
Supplement: Supplemental Information 1 [file peerj-12-16613-s001.pdf]

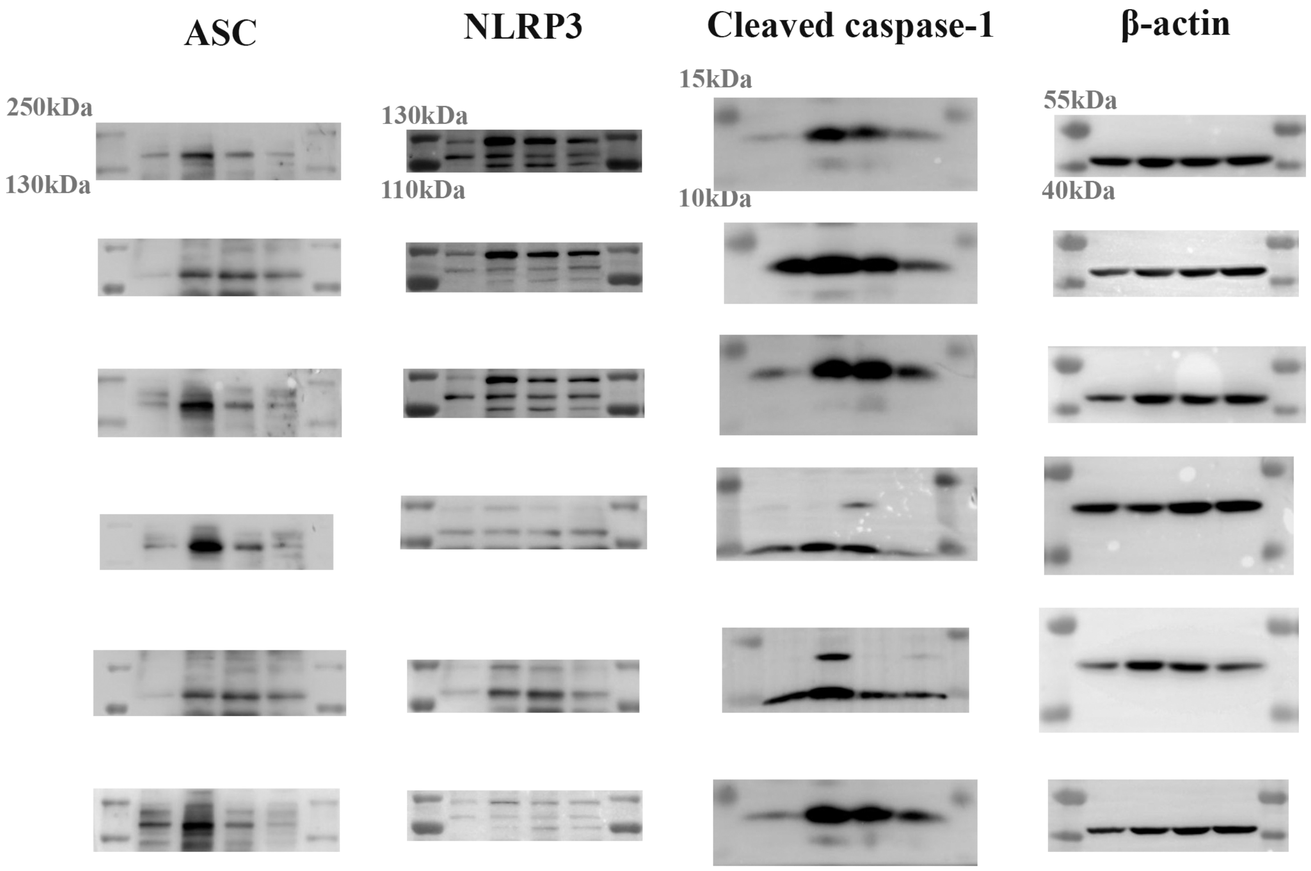

the group of lanes in the pictures from left to right is control, MIRI, MIRI + low ticagrelor, MIRI + high ticagrelor;

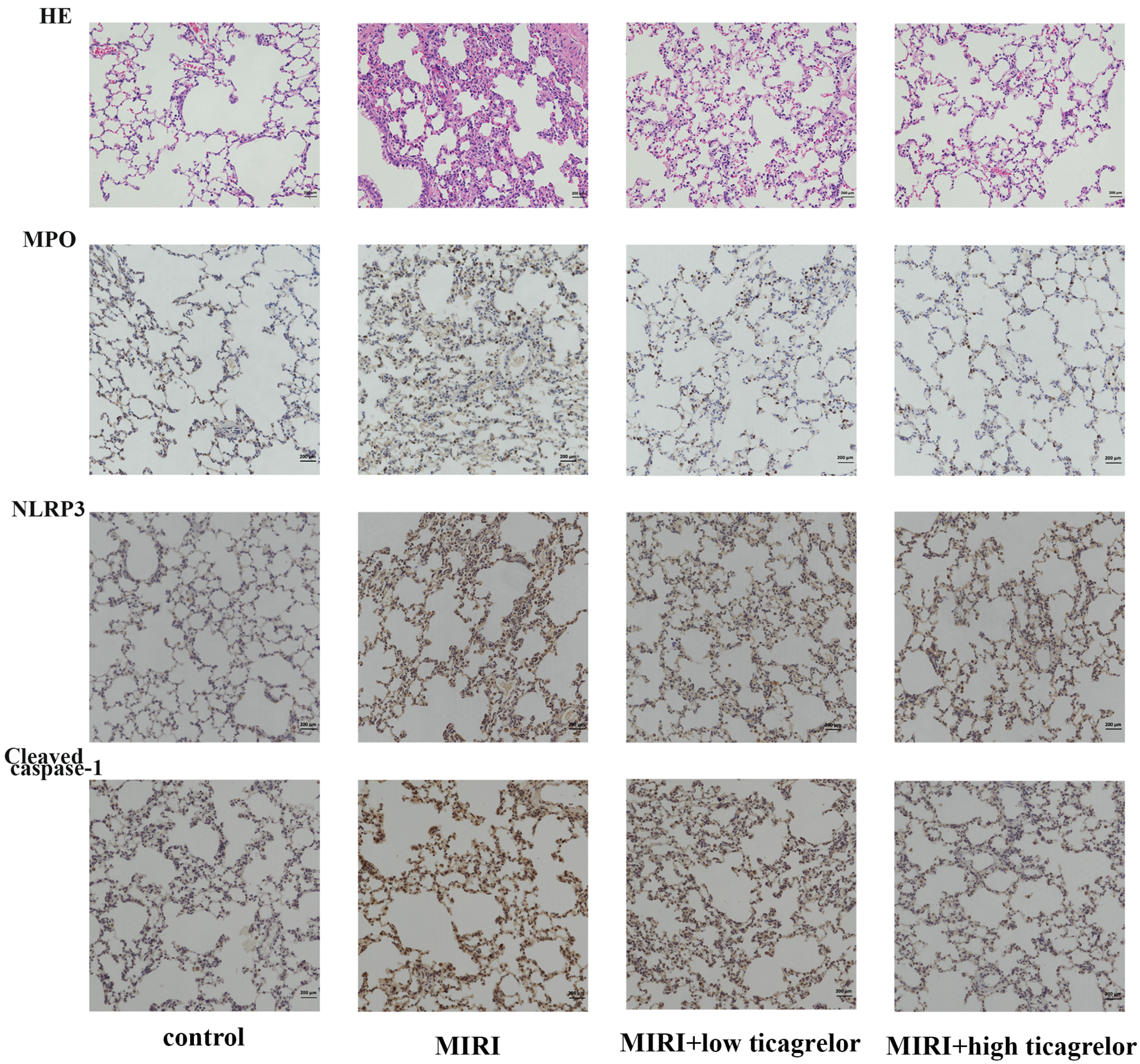

HE
MPO
NLRP3
Cleaved caspase-1
control
MIRI
MIRI+low ticagrelor
MIRI+high ticagrelor
